# Traditional microscopy instruction versus process-oriented virtual microscopy instruction: a naturalistic experiment with control group

**DOI:** 10.1186/1746-1596-6-S1-S8

**Published:** 2011-03-30

**Authors:** Laura Helle, Markus Nivala, Pauliina Kronqvist, Andreas Gegenfurtner, Pasi Björk, Roger Säljö

**Affiliations:** 1 University of Turku, Centre for Learning Research, 20014 Turun yliopisto, Finland; 2 University of Turku, Department of Pathology, 20014 Turun yliopisto, Finland; 3 University of Gothenburg, Department of Education, P.O. Box 300, 40530 Gothenburg, Sweden

## Abstract

**Background:**

Virtual microscopy is being introduced in medical education as an approach for learning how to interpret information in microscopic specimens. It is, however, far from evident how to incorporate its use into existing teaching practice. The aim of the study was to explore the consequences of introducing virtual microscopy tasks into an undergraduate pathology course in an attempt to render the instruction more process-oriented. The research questions were: 1) How is virtual microscopy perceived by students? 2) Does work on virtual microscopy tasks contribute to improvement in performance in microscopic pathology in comparison with attending assistant-led demonstrations only?

**Method:**

During a one-week period, an experimental group completed three sets of virtual microscopy homework assignments in addition to attending demonstrations. A control group attended the demonstrations only. Performance in microscopic pathology was measured by a pre-test and a post-test. Student perceptions of regular instruction and virtual microscopy were collected one month later by administering the Inventory of Intrinsic Motivation and open-ended questions.

**Results:**

The students voiced an appreciation for virtual microscopy for the purposes of the course and for self-study. As for learning gains, the results indicated that learning was speeded up in a subgroup of students consisting of conscientious high achievers.

**Conclusions:**

The enriched instruction model may be suited as such for elective courses following the basic course. However, the instructional model needs further development to be suited for basic courses.

## Introduction

**Virtual microscopy** is being introduced in medical education as an approach for learning how to interpret information in microscopic specimens. The basic idea is that the specimens can be viewed via the Internet from one’s computer screen instead of using a light microscope. Thus, inspection of the slides is no longer restricted to tutorials: students can view images of the slides at any time from almost any computer with an Internet connection. This, in turn, increases opportunities for using instructional approaches that rely on more independent, project-oriented work among students. Virtual microscopy also has several practical advantages, including “economies of scale.” Hundreds of students can view the same picture, and this eliminates the need to produce sets of glass slides for large student cohorts. Even more importantly, groups of students can view exactly the same specimen allowing for the same point of reference in group work and discussions as well as in exam situations.

**Figure 1:**
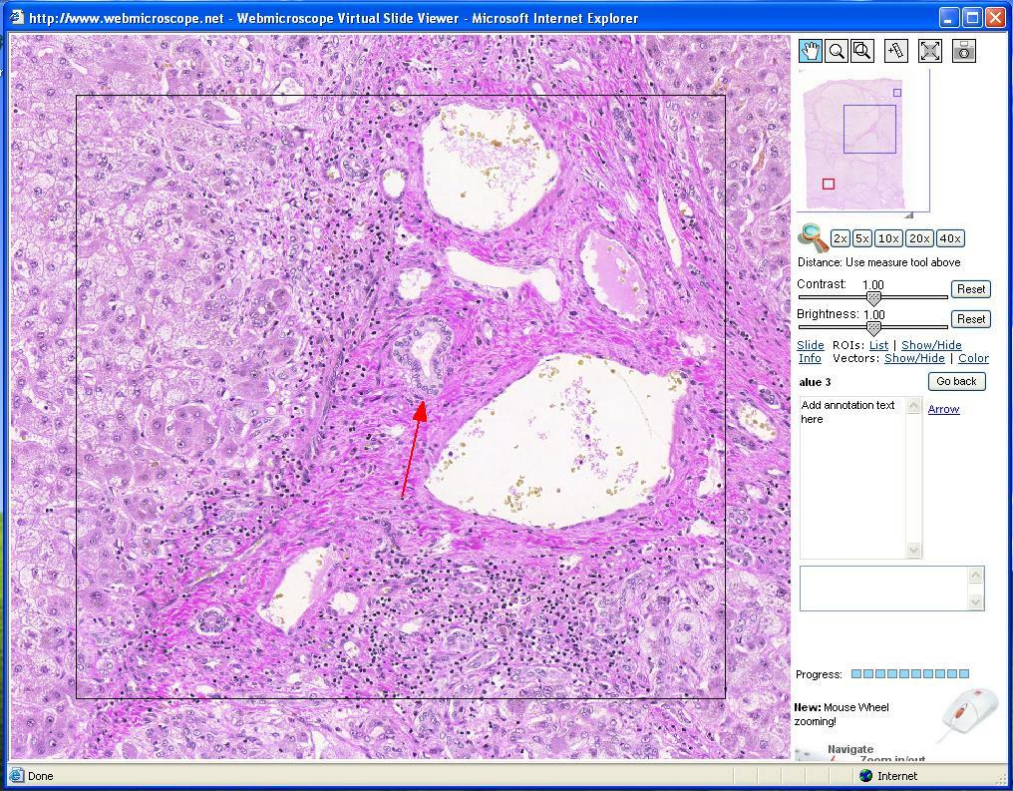
A WebMicroscope view

A group of Finnish specialists in medical informatics has designed a virtual microscopy application for instructional purposes, which is called the WebMicroscope [1,2]. As illustrated in Figure 1, one can manipulate the image on one’s computer screen by zooming in and out of the virtual slide displayed in the upper right hand corner, and one can adjust for contrast and brightness. Furthermore, one can mark certain areas and save one’s comments regarding those areas (“annotations”). The annotations can be used by the student or by the teacher for didactic purposes. Thus, the interactivity between the student and the specimen is high, as recommended by experts [3,4], and contains new elements (figure 1).

It is, however, far from evident how to incorporate virtual microscopy into existing teaching practice. Traditionally, teaching of undergraduate microscopic pathology has consisted of theoretical lectures and assistant-led microscopy demonstrations. In order to devise solutions, the local department of pathology has entered a multi-year collaboration with the local Centre for Learning Research. In consequence, a multidisciplinary group is experimenting with ways of integrating the WebMicroscope into the teaching and learning of pathology.

The adoption of new technology provides an opportunity for the users and developers to reanalyze and reshape teaching practice [5]. In fact, Vesisenaho has cautioned against merely importing technology without prior needs analysis of local conditions [6]. In this case, an analysis of teaching practice led to complementing traditional teaching with elements of *process-oriented* instruction.

### Process-oriented instruction vs. traditional instruction

In the medical domain, traditional instruction in many fields, such as pathology, relies heavily on the learning of lists of symptoms relating to dozens of diseases. As Norman et al. [7] noted, medical students in traditional medical schools “spend endless hours learning the 29 causes of anemia or the signs and symptoms of Hashimoto’s disease.” It is argued that this approach suffers from certain shortcomings. First, the amount of detail puts a great burden on the memory capacity of students. Second, when learning attempts fail, students appear lost. Third, this instructional approach may not be optimally conducive to intrinsic motivation. Activity is intrinsically motivating when it is perceived as interesting and enjoyable. The process-oriented model designed for the present study represents an effort to overcome the shortcomings of didactic instruction. **Process-oriented instruction** refers to teaching, in which the teacher engages in interaction with the students regarding subject matter, assigns tasks to students and gives instructions regarding the learning process [8].

**Figure 2:**
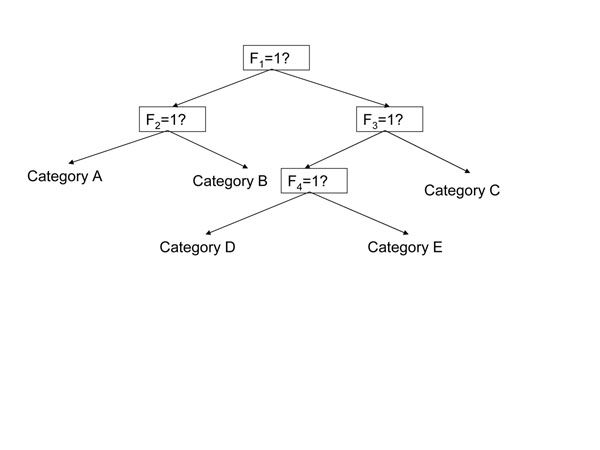
An example of a classical decision-tree.

According to van Merriënboer and Sweller [9], the most commonly used method to help students in rich learning tasks requiring problem solving and reasoning skills is to provide them with some kind of **process worksheet**. See also [10,11]. A process worksheet provides a generic, but content-based, description of the phases one should go through when solving a problem. It may also provide hints or rules-of-thumb that help students complete each successive phase. The present study follows a process worksheet approach based on a decision-tree, which is a tree-structured classifier containing test nodes and categorization nodes [12]. A test node indicates a feature test to be carried out, whereas a categorization node (i.e. an exit node) indicates the value of the classification (i.e. the final result). The obvious advantage of decision-trees is that they can overcome the limitations of propositional rules by embedding them within an explicit serial decision process. An example of a classical decision-tree is shown in Figure 2. It follows the convention that the branches to the lower left of a test node (indicated by a box) indicate a “yes,” whereas the branches to the lower right indicate “no.” Exit nodes indicate the final answer (figure 2).

The purpose of the present naturalistic experiment is to explore the consequences of introducing virtual microscopy into undergraduate pathology instruction in an attempt to render the instruction more process-oriented. More specifically, the research questions are:

1) How is virtual microscopy perceived by students?

2) Does work on virtual microscopy tasks contribute to improvement in performance in microscopic pathology in comparison with attending assistant-led demonstrations only?

## Methods

### Participants

The participants (N = 120) were second-year medical students from a Finnish faculty of medicine attending a four-month (10-ECTS) undergraduate course in pathology that consisted of theoretical lectures, demonstrations in microscopy in a lecture hall, assistant-led demonstrations in microscopy, and participation in a set of autopsies and seminars. The demonstrations in microscopy took place during a six-week period at the beginning of the course and provided the context for the present study. The objective of the undergraduate course in pathology is to convey an understanding of how changes at tissue and cellular level impact health and disease. The microscopy instruction aims at teaching diagnostic classification based on microscopic observation. For the study of affective outcomes, there were 93 respondents. For the study of learning effectiveness, there were 54 participants.

These 54 students had participated in a pre-test (version A) and a post-test (version A). Pre-test version (B), which was equivalent to post-test version A, had a low reliability because it was too difficult for beginners, resulting in having to discard approximately half of the data. Therefore, the number of participants in the learning effectiveness study was much lower than anticipated.

### Design

**Figure 3:**
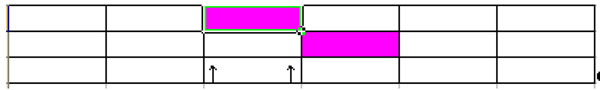
Design of the naturalistic experiment during the microscopy instruction period

Within approximately one week in the middle of the microscopy instruction period, half of the participants were asked to complete three sets of homework assignments pertaining to three different systems (of human organs) to be done in pairs. The other half of the students served as controls (no assignments). In order to assure equal opportunities for all, the controls received virtual microscopy assignments the following week. The design is presented in Figure 3. The rectangles indicate approximately one week of instruction in microscopic pathology. The purple rectangles refer to weeks of enriched instruction, whereas the white rectangles indicate weeks of instruction as usual. The arrows indicate the timing of the pre- and post-test. As mentioned earlier, there were two partially overlapping versions of the pre-test (A and B) and the post-test (A and B), but only version A proved reliable (figure 3).

### Instructional procedure

The instructional intervention was based on two design principles: 1) More gradual shift from teacher-regulated instruction to self-study, 2) Elements of process-oriented instruction such as the assignment of tasks, feedback, and the use of process worksheets. It consisted of the following elements:

1. Self-paced practice in pairs on distinguishing critical abnormal features (use of teacher-prepared annotations to visualize zones of interest) and practice on making a diagnosis

2. Decision-tree to visualize the process of making a diagnosis

3. Collective feedback to students

**Figure 4:**
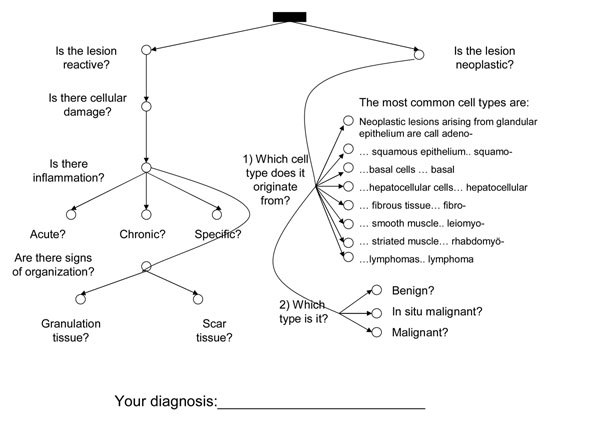
Process worksheet displaying the decision-tree

The homework and the process worksheet were delivered by the WebMicroscope. The process worksheet, which was first introduced to the students during the pre-test, was in the form a decision-tree. The first test node in the decision-tree represented the decision regarding whether the case represented a case of neoplastic or reactive disease (see Appendix 1, Figure 4). After this critical decision, further steps were provided. A simplified version of the decision-tree was provided, so that students could mark intermediate decisions leading to the final diagnosis. A “yes” was to be marked by a cross in the circle next to each question. It is worth noting that since the undergraduate pathology curriculum covers approximately two hundred different diseases, not all of the exit nodes could be explicitly stated.

The homework tasks consisted of three parts following a simple-to-complex ordering strategy (i.e. isolated elements are presented first and only afterwards the whole with its interacting components [13]). In Part I, the students were presented with certain areas of the slide that were highlighted by a circle, an arrow, or another mark and asked to select the correct answer from a list of alternatives. In Part II, the students were once more presented with annotated areas, but this time asked to describe the findings. In Part III, the students were asked to suggest a diagnosis for a slide without annotations. Each assignment was to be completed before an assistant-led demonstration session. The assistant was expected to go over the assignments before commencing teaching.

After the intervention period, fully annotated digital versions of the example cases shown in the assistant-led demos were made available to the students.

### Materials and procedures

The pre- and post-test materials consisted of 1) ten partially overlapping multiple-choice questions per student asking the student to identify a particular histological abnormality (i.e. a single feature), and 2) six questions asking the student to suggest a diagnosis. For the features test, one point was given for each correct answer. As for the diagnosis test, one point was given if the student had arrived at the correct general diagnosis (neoplastic or reactive). A pre-test composite index was formed of the average 11 items and a post-test score of the average of 11 items partially overlapping with the pre-test. Items included in the pre-test composite index are marked with an asterisk (*) and items in post-test with a hash (#) in Tables 1 and 2 in Appendix 2. The Kuder-Richardson coefficient of reliability (K-R 20) recommended for dichotomous variables was 0.64 for the pre-test and 0.61 for the post-test. According to Nunnally & Bernstein, the use of somewhat modest reliabilities can be justified in exploratory research [14]. See also [15,16].

**Table 1:** Results from test of feature classification.

Item		Pre		Post	
No.	Content	n	% correct	n	% correct
1A*	Epidermal ridge	59	14	50	76
2A*	Parakeratosis	59	54	50	56
3A	Neutrophil granulocyte	59	5	50	8
4A*	Skin appendages	59	42	50	58
5A*	Thyroid follicle	59	75	50	82
6A*#	Colloid	114	33	105	55
7A*	Epithelial hyperplasia	114	1	105	17
8A#	Lymphocyte aggregation	114	61	105	69
9A*#	Normal thyroid	114	33	105	41
10A*#	Papillary structures	114	44	105	86
11A	Psammoma body	55	62	55	95
12A#	Nuclear groove	55	31	55	80
13A#	Lymphoid germinal centre	55	66	55	87
14A#	Mitosis	55	60	55	67
15A#	Oncocyte	55	0	55	9

(Items marked by an asterisk (*) are part of the pre-test composite index, and items marked by a hash (#) are part of the post-test. The respondents of A1–A5 and A11–A15 consist of non-overlapping samples.)

**Table 2:** Results from test of diagnostic classification.

Item		Pre		Post	
No	Content	N	% correct	n	% correct
1B	Ulcer	59	49	50	42
2B*	Basal cell carcinoma	59	97	50	96
3B*	Invasive ductal carcinoma of the breast	59	95	49	98
4B*	Intraductal carcinoma of the breast	114	81	105	94
5B	Fibroepithelial polyp	114	77	105	72
6B#	Prostatitis	114	54	105	53
7B#	Crohn’s disease	55	55	55	60
8B	Metastatic adenocarcinoma	55	84	55	89
9B#	Hepatocellular carcinoma	55	47	55	56

(Classification is judged to be “correct” if the case is diagnosed correctly as neoplastic or reactive. The respondents of B1–B3 and B7–B9 consist of non-overlapping samples.)

Approximately one month later, data were gathered on student perceptions regarding the intervention vis-à-vis regular instruction. Students were asked to complete the *Intrinsic Motivation Inventory* (IMI: [17]) comparing the two instructional conditions on a seven-point Likert scale. Each item had two variants: the one referred to virtual microscopy assignments and the other to regular microscopy demonstrations. For reliabilities and sample items, please see Table 3 in Appendix 3. Students were also asked for written comments on the intervention.

**Table 3:** Motivational constructs from the Intrinsic Motivation Inventory (IMI), sample items and psychometric properties (N = 93). (Figures above refer to the virtual microscopy condition and figures below to ordinary instruction.)

Construct	Example item	Number of items	Cronbach Alpha
Interest/enjoyment	“I would describe this instructional intervention (W) /the microscopic demonstrations (N) as very interesting.”	7/7 (all original IMI items)	0.85 0.90
Perceived competence	“I think I was pretty good at W/N.”	6/6 plus one additional item	0.84 0.90
Perceived effort	“I put a lot of effort into W/N.”	5/5 (all original items)	0.82 0.86
Pressure/tension	“I felt very tense while doing W/N.”	5/5 (all original IMI items)	0.74 0.83
Perceived choice	“I did W/N because I had to.” (reversed)	5/7 IMI items	0.76 0.90
Value/usefulness	“I think doing W/N was useful for my learning.”	7/7 IMI items plus one additional item	0.86 0.90

### Analysis of open comments

The open, written comments of the students were transcribed (two pages) and grouped under the following five rubrics: positive comments regarding the virtual microscopy experiment, negative comments regarding the experiment, feedback/suggestions, technical aspects, and feedback on the questionnaire.

### Statistical analysis

The motivational items from the IMI were analyzed with paired-samples (t-tests) as each item had two variants.

The learning results were first analyzed descriptively by simply comparing the means and modes of the responses on the post-test with those of the pre-test. As the pre-test and the post-test contained a partially overlapping set of items, comparing pre-test and post-test scores on absolute terms would not have made sense. Instead, we examined a possible interaction effect using repeated-measures ANOVA. The repeated-measures within-subjects factor was time (pre-test composite index/post-test composite index) and between-subjects factor was group (experimental/control). As it was *presumed* that high achievers would be more likely to be able to make use of the scaffolds provided, a further analysis (ANCOVA) was conducted on high and low achievers separately. ANCOVA was used in order to determine if the experimental group would outperform the control group on the post-test when controlling for the level of perceived effort as the level of perceived effort differed in the two groups (experimental/control). There was no difference between the two groups in the performance on the pre-test.

## Results

### Student perceptions of virtual microscopy

There were many positive (N = 20) comments provided by the students. Five of the positive comments concerned either independent study or studying from home, e.g., “Having a virtual specimen is really good; it is easy to study at home.” According to three comments, the annotations were seen as useful, e.g., “The annotations were fantastic. Some cell types and phenomena can be made clearer.” The significance of annotations is also clear from the constructive suggestions by the students: in thirteen comments, more annotations were requested. Three comments pertained explicitly to the virtual homework assignments. The message was that they helped *prepare* for the assistant-led demos, e.g., “Virtual homework should be provided on a continual basis; they helped me prepare and look things up before the assistant-led demo. Then it was easier to ask things that remained unclear.” Two comments reflected on the complementary nature of assistant-led demos and virtual microscopy. Two students praised the software, both using the adjective “fantastic.” One described the decision-tree as a “good innovation.”

The negative/constructive comments were mainly technical in nature (N =11), which was hardly surprising as the technology was still in the making. Typical complaints were that annotations did not appear (N = 3) or the software was incompatible with the operating system or the browser (N = 4). One comment related to the fact that it would be helpful to be able to view several specimens at one time in order to compare, e.g., normal and abnormal tissues. The negative or constructive comments related to instruction mainly had to do with the stifling nature of the virtual microscopy tasks (N = 3). One commentator, however, expressed that microscopy as such is stifling (“Both regular and virtual microscopy are stifling and cause headaches although I see that they are important”). Other complaints included perceived task difficulty (N = 2), a lack of feedback in assistant-led demos (N = 2), and timing (N = 3), e.g., “It would be better to do virtual microscopy homework tasks on tissues that have been taught during the lectures” or “the virtual homework tasks should be made available earlier.” One comment related to the need to develop the decision-tree further. However, one message (N = 13) stood above everything else: “More annotations!”

Although it is clear from the student comments that a comparison between virtual microscopy (assignments) and teaching is not relevant as they can be viewed complementary, the results on the comparison between motivation in teacher-led microscopy demonstrations and virtual microscopy homework tasks are presented in Table 4. As could be expected, pressure was higher in the teacher-led condition (M=5.4 SD=1.05) compared to the virtual microscopy condition (M=4.93 SD=1.03) than in the homework condition. In addition, the perceived level of effort was higher in class (M=4.36 SD=1.06) than doing virtual microscopy homework (M=3.46 SD=0.99). In contrast, there was no statistically significant difference in the experience of interest/enjoyment (i.e. intrinsic motivation proper) and perceived choice. Teacher-led demonstrations were rated as somewhat more useful for learning (M=5.73 SD=0.83) than virtual microscopy homework tasks (M=5.01 SD=0.89). For a similar result, see Haidet et al. [18] (Figure 4).

**Table 4:** Elements of student motivation in normal instruction (figure above) and virtual microscopy (figures below) (N = 93) (Scale 1-7).

	M	SD	Sign
Interest/enjoyment	4.84	1.01	
	4.99	0.87	n.s.
Perceived competence	4.59	0.97	
	3.68	0.96	p < 0.001
Perceived effort	4.36	1.06	
	3.46	0.99	p < 0.001
Pressure/tension	5.42	1.05	
	4.93	1.03	p < 0.001
Perceived choice	5.25	1.17	
	5.08	0.87	n.s.
Value/usefulness	5.73	0.83	
	5.01	0.89	p < 0.001

### Learning results

Tables 1 and 2 provide an overview of student performance on the pre- and post-test. As a trend, students’ diagnostic classification of individual *features* improved during the one-week period, but diagnostic classification of whole cases, which is a higher-order skill, did not improve.

As for the effect of the intervention, an initial analysis using repeated-measures analysis of variance revealed no difference in the development of the performance of the experimental and control group. However, when limiting the analysis to the higher achieving half of the students based on pre-test score (N = 20) who had participated in the pre-test, post-test, and motivational measurement, the experimental group outperformed the control group (M_ex_= 0.79 SD=0.13; M_c_=0.69 SD=0.15). The difference was statistically significant (*F*(1)=4.3, one-tailed *p*=0.027). The difference was even more pronounced when controlling for perceived effort Mex= 0.80 s.e.=0.05; Mc=0.66 s.e.=0.04) and statistically significant (*F*(1)=4.5, one-tailed *p*=0.024). What about the low achievers? There was not enough data on the low achievers to answer this question as their participation in the measurement sessions was much less regular than the participation of the high achievers.

## Discussion

Based on retrospective reports, the students saw the value of virtual microscopy for the course and individual study. Naturally, there were some uncertainties as this was the students’ and the faculty members’ first experience with virtual microscopy and the students were not accustomed to homework. It is well known that the integration of state-of-the-art educational technology into a real world setting does not occur over night, but requires time [19]. It was in fact feared by faculty members that homework might be resented by the students, which according to the results of the present study was not the case. As for learning gains, the results indicated that learning was speeded up in a subgroup of students consisting of conscientious high achievers.

This result, and the failure to obtain the desired results in the larger group, indicate that further measures to decrease cognitive load in virtual microscopy have to be considered seriously. For example, the participants were subjected to extrinsic cognitive load by presenting supplementary and process information during the performance situation. Van Merriënboer and Sweller [8] have suggested that cognitive load can be optimized by presenting supplementary information before and process information during learning. In addition, the fact that the homework assignments contained new information may have caused too much cognitive load for some students.

There are some limitations to the study. Due to the small number of participants, it cannot be ruled out that the ANCOVA results could have been due to chance. To minimize this possibility, the prerequisites for using ANCOVA were examined with great care: a) errors should be normally distributed and homoscedastic; b) variances should be equal in each group; c) regression should be linear (between covariate and dependant variable); d) homogeneity of regression (relationship between the covariate and the dependent variable should be similar across all groups of the independent variable) [20]. No deviations to these assumptions could be detected. Thus, the result can be taken as *tentative* evidence suggesting the implementation of the process-oriented approach may be viable, at least for conscientious students with a certain threshold of pre-existing knowledge. We also believe that extending the period of process-oriented learning from one week to several of weeks and making it an integral element of instruction instead of an experimental add-on would make the effects stronger.

We have learned many valuable lessons along the way. The decision-tree is currently under further development. We are also developing a more systematic step-wise approach to incorporate the process worksheet into instruction. The approach is believed to be ideal for undergraduate courses since it focuses attention on certain key concepts instead of dispersing attention to hundreds of symptoms of different diseases. A vision for the future could be to reduce the number of cases studied at the undergraduate level (currently approximately two hundred) to a smaller number of “exemplary” cases [21], which would allow even higher interactivity between students, teachers, and materials conducive to high quality, active learning.

## Conclusions

The theoretical contribution of the study is that it describes a fresh, innovative approach to the instruction of microscopy. The decision-tree approach builds on state-of-the-art findings from research on instructional design. The practical implication is that the enriched instruction model may be suited as such for elective courses following the basic course. However, the instructional model needs further development to be suited for the basic courses.

## Competing interests

The authors declare that they have no competing interests.

## Authors’ contributions

LH coordinated the conception, design and analysis the study, participated in the collection of the data and drafted the manuscript. MN participated in the conception and design of the study. He also participated in data collection. As content-specialist, PK participated in designing materials for testing and training purposes. In addition, she had an active role in the conception and design of the study. AG contributed to the design of the study, data collection and revision of the manuscript. PB participated in data collection and analysis. RS participated in the design of the study and help to draft the manuscript. All authors have read and approved the final manuscript.

## Notes on contributors

Dr Helle is a senior researcher in a project called LearnMedImage funded by the Acadeny of Finland. She has a research interest in learning and professional development in complex domains including medicine.

Mr Nivala is a PhD student in the LearmedImage project. His research interests include educational technology and development of visual diagnostic competence in medicine.

Dr Kronqvist is clinical teacher of pathology and adjunct professor of experimental pathology at the University of Turku. She is an inspiring teacher and she has received several of awards for her work in developing pathology instruction.

Mr Gegenfurtner is a PhD student in the LearnMedImage project. His research interests include the development of visual expertise, motivation, and professional training.

Mr Björk is completing his master’s level studies in teacher education. His interest to work in the project stemmed from his studies in medicine.

Dr Säljö is professor of education and educational psychology at the Department of Education, University of Gothenburg and Finnish Distinguished Professor at the University of Turku. He is the leader of the LearnMedImage project.
